# Flame-Retardant Mechanism of Layered Double Hydroxides in Asphalt Binder

**DOI:** 10.3390/ma12050801

**Published:** 2019-03-08

**Authors:** Kai Zhu, Yunhe Wang, Daquan Tang, Qiang Wang, Haihang Li, Yadong Huang, Zhiyi Huang, Ke Wu

**Affiliations:** 1 College of Quality and Safety Engineering, China Jiliang University, Hangzhou 310018, China; zhukai@cjlu.edu.cn (K.Z.); p1706085225@stu.cjlu.edu.cn (Y.W.); TDQ_1127@163.com (D.T.); qiangwang@cjlu.edu.cn (Q.W.); lihaihang@cjlu.edu.cn (H.L.); 2 College of Civil Engineering and Architecture, Zhejiang University, Hangzhou 310058, China; hzy@zju.edu.cn; 3 Fire Bureau of Zhejiang Province, Hangzhou 310014, China; yadong_huang@163.com; 4 Key Laboratory of Offshore Geotechnics and Material of Zhejiang Province, Zhejiang University, Hangzhou 310058, China

**Keywords:** asphalt binder, layered double hydroxides, flame-retardant mechanism, cone calorimeter, char layer

## Abstract

The flame retardancy of asphalt binders with layered double hydroxides (LDHs) was investigated using limiting oxygen index (LOI) and cone calorimeter tests. The flame-retardant mechanism of the LDHs was also studied with thermogravimetry and differential scanning calorimetry (TG–DSC), scanning electron microscopy (SEM), and X-ray photoelectron spectroscopy (XPS). The cone calorimeter testing results indicated that 2 wt.% of the LDHs can decease the peak heat and smoke release rate of asphalt binders. Because a low dose of LDHs can be well dispersed in asphalt binder and favor the formation of polyaromatic structures during combustion, the thermal oxidation resistance and compactness of the char layer can be improved. The LOI of asphalt binder can be increased and the heat and smoke release during combustion can be decreased with 25 wt.% LDHs. The decomposition of LDHs can absorb the heat release of the initial two stages of asphalt combustion and reduce the burning rate of asphalt. Due to the loss of loosely bound water in the LDHs during the blending process and the decrease of dispersibility at a high LDH dose, the improvement of thermal stability is limited.

## 1. Introduction

Asphalt is widely used as a binder in pavement, waterproof, pipeline antirust, etc. [1]. However, asphalt is flammable and can be easily ignited during irradiation and convection heating with fire [2]. In particular, in a tunnel fire, the temperature can exceed 1000 °C in a few minutes [3]; the ignited asphalt pavement then releases toxic fumes and heat [4,5], seriously endangering the safety of persons in tunnels and hindering their rescue.

A common method to improve the fire resistance of asphalt binder is to add flame retardants [6,7,8,9,10,11]. Notorious pollutants that arise from thermal decomposition of the commonly used brominated flame retardants (BFRs) [12,13,14] limit their use in flame-retardant asphalt. Due to their advantages of environmental friendliness and smoke suppression, metal hydroxides, i.e., magnesium hydroxide (MH) [11,15,16] and aluminum hydroxide (ATH) [17,18,19], have gradually replaced BFRs for asphalt. Metal hydroxides decompose at temperatures close to the ignition point of asphalt and absorb heat from combustion, consequently lowering the temperature of the asphalt binder. At the same time, the released water vapor dilutes the concentration of flammable volatiles, and the residues (MgO, Al_2_O_3_) show a strong charring effect, which can form a film to prevent contact between oxygen and the asphalt binder and elevate the flame retardancy of the asphalt materials [15,16]. However, these commonly used metal hydroxides (MH/ATH) reduce the low-temperature crack resistance of asphalt [6] and affect the pavement performance of asphalt concrete.

Layered double hydroxides (LDHs) are a family of hydrotalcite compounds with multinested layered structures consisting of positively charged laminates and interlayered anions [20,21]. Asphalt with LDHs can show improved low-temperature performance compared to base asphalt [21]. Moreover, LDHs can also improve the aging resistance of asphalt under both thermo-oxidative aging and ultraviolet (UV) aging [20]. The antiaging mechanism can be due to the multilayered structure of LDHs not only blocking the oxygen but also reflecting the high-energy UV light at the interface of the LDHs sheets [22,23,24]. The above antiaging mechanism can also play a certain role in blocking oxygen diffusion and shielding radiation during asphalt combustion.

LDHs have been used as flame retardants for polymers because they combine the features of both conventional metal hydroxides, such as MH or ATH, and nanoclays, such as sepiolite or montmorillonite [25]. By adding LDHs, the thermal stability of polypropylene (PP), poly (methyl methacrylate) (PMMA), polyethylene (PE) and polystyrene (PS) can be improved, and the peak heat release rate (PHRR) of these polymers can also decrease [26,27]. It should be noted that the flame retardancy of LDHs is significantly affected by the polymer type. For example, ZnAl–LDHs lead to a large improvement in the thermal stability of PE, whereas MgAl–LDHs enhance the thermal stability of PMMA, which is a more polar polymer [26]. Asphalt is a complex mixture of different constituents, including hydrocarbons and hetero-atoms [28], and it is quite different from the polymers discussed above. However, investigations into the thermal stability and flammability of asphalt binders with LDHs are still limited. Li et al. [29] investigated the effect of ATH and LDHs on the flame retardancy and smoke suppression of asphalt with limiting oxygen index (LOI) testing and thermogravimetry–differential scanning calorimetry–mass spectrometry (TG–DSC–MS), and good synergistic effects of ATH and LDHs were reported. However, the flame-retardant mechanism of LDHs in asphalt binder remains unclear.

In the present study, the flame retardancy of asphalt binder with LDHs was investigated using an LOI analyzer and cone calorimeter, and the flame-retardant mechanism of LDHs was studied with TG–DSC, scanning electron microscopy (SEM), and X-ray photoelectron spectroscopy (XPS). The results of this study can provide a scientific basis for the application of LDHs in flame-retardant asphalt binders.

## 2. Materials and Methods

### 2.1. Materials

A base asphalt (BA) of PG 64–22 was used, which was provided by Jiangsu Baoli International Investment Co., Ltd., Wuxi, China. The basic properties of the asphalt binder are shown in Table 1. ZnMgAl–CO_3_–LDHs were applied to base asphalt as a flame retardant, which was provided by Shandong Vansinvena Material Technology Co., Ltd., Linyi, China, and is a white powder with a specific surface area of 13.2 m^2^/g and a purity of 99.0%.

With a low dose of LDHs (2 wt.%), the antiaging performance of asphalt was improved [22,23,24], and flame-retardant effects were also reported for PP, PMMA, and PS [26,27]. Therefore, 2 wt.% was chosen as the minimum dose of the LDHs, and the barrier mechanism of the LDHs in asphalt combustion was the main focus. With a high dose of metal hydroxide (25 wt.%), an excellent flame-retardant effect for asphalt by the decomposition reaction was exhibited [11,15,16,17,18,19]. Therefore, 25 wt.% was chosen as the maximum dose of the LDHs, and the decomposition endotherm mechanism was the main focus.

The flame-retardant asphalt was prepared by melt blending. First, asphalt binder was loaded into a stainless-steel canister and heated to 160 ± 5 °C by a sand bath. Then, the asphalt was stirred at a rate of 1000 rpm by a mixing machine for 15 min while LDHs were added into the asphalt binder. Then, the mixture was stirred with a BME100LT high-shear machine (Edong Electrical and Mechanical Equipment Co., Ltd., Shanghai, China) at a rate of 5000 rpm for 30 min to ensure a uniform distribution of the LDHs. Finally, the mixture was stirred for an additional 15 min at a rate of 500 rpm to eliminate air bubbles. Four doses of LDHs were used, including 2 wt.%, 5 wt.%, 10 wt.%, and 25 wt.%, and the asphalt binders are referred to as AL2, AL5, AL10, and AL25, respectively.

### 2.2. Test Methods

The X-ray diffraction (XRD) patterns were obtained using an X’Pert3 Powder diffractometer (Malvern PANalytical, Almelo, Netherlands) with Cu Kα radiation (*λ* = 1.5406 nm) at 40 kV and 40 mA. The 2*θ* scan range for LDHs and asphalt binders was from 10° to 70°.

The surface morphologies were obtained by a SU8010 field emission scanning electron microscope (Hitachi, Ibaraki Prefecture, Japan). The specimens were previously spray-coated with a thin platinum layer to improve conductivity before SEM observation. The elemental analysis of the asphalt binders was investigated by SEM with an X-Max^N^ energy-dispersive spectrometer (EDS, Oxford Instruments, Oxford, UK) at an accelerating voltage of 20.6 kV.

The LOI test was performed on a HC-2 LOI analyzer (Jiangning Analysis Instrument Co., Ltd., Nanjing, China) according to ASTMD-2863-77. Since the asphalt binder melts and flows after ignition, the sample was supported by the glass filter felt. For the sample preparation, 3 pieces of 150 mm × 150 mm glass filter felt were cut, placed on a glass plate coated with asphalt release agent, and the asphalt binder was heated to 160–170 °C. Next, 75 g (approximately 25 times the mass of the felts) of asphalt was poured uniformly on the glass filter felts. After the sample was cooled to room temperature, it was cut into samples that were 120 mm in length and 6.5 ± 0.5 mm in width.

The cone calorimetry tests were performed using a 007 cone calorimeter (Fire Testing Technology, London, UK) according to ISO 5660-1:2002 standard procedures under a heat flux of 50 kW/m^2^. The sample was poured into a 100 mm × 100 mm dish with a thickness of 5 mm and wrapped in aluminum foil. The experiments were performed three times under each condition, and the average value was calculated.

A STA 449 F5 Jupiter^®^ thermogravimetry and differential scanning calorimetry instrument (NET-ZSCH, Selb, Germany) was used to study the thermal properties of the asphalt binders. Approximately 5 mg of the sample was placed in an aluminum oxide crucible. The sample was heated from 40 °C to 650 °C at a heating rate of 10 °C/min under air atmosphere with a gas flux of 40 mL/min. Before the experiments were conducted, a temperature and balance calibration was performed, and the results showed that the measurement repeatability was satisfactory.

The asphalt binder residues after cone calorimetry tests were analyzed by X-ray photoelectron spectroscopy (XPS) on an ESCALAB Mark II spectrometer (VG Scientific, Waltham, UK) with Mg Kα radiation (*hν* = 1253.6 eV).

## 3. Results and Discussion

### 3.1. Structure and Morphology Characterization

**Figure 1:**
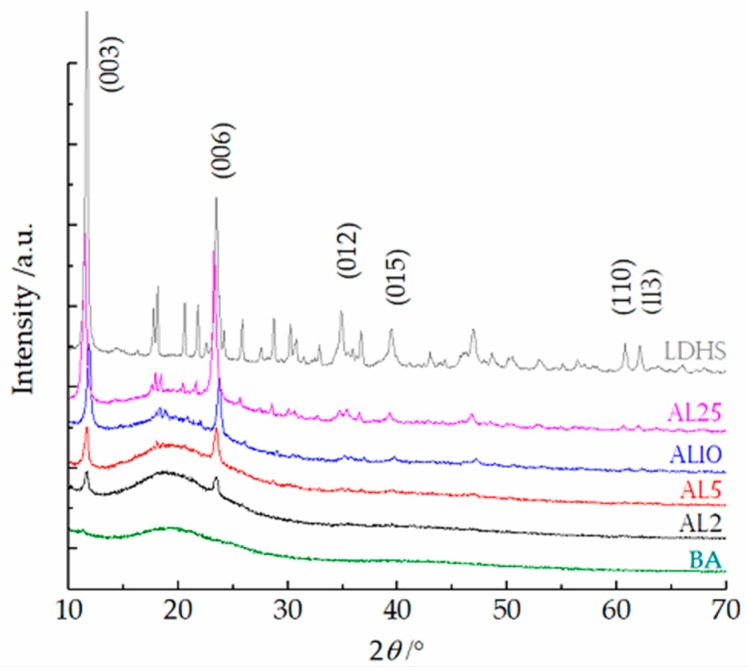
XRD patterns of base asphalt (BA), AL2 (asphalt/2 wt.% LDHs), AL5 (asphalt/5 wt.% LDHs), AL10 (asphalt/10 wt.% LDHs), AL25 (asphalt/25 wt.% LDHs), and LDHs.

The XRD patterns of LDHs powder and asphalt binders with different LDH doses are shown in Figure 1. It can be seen from the XRD patterns that there are two sharp and strong characteristic peaks at low 2*θ* angles, corresponding to diffraction by the (003) planes (*d*_003_ = 0.76 nm) and (006) planes (*d*_006_ = 0.38 nm). There are also two relatively weak peaks at high 2*θ* angles (60°–70°), corresponding to diffraction by (110) planes and (113) planes. When the LDHs were added to asphalt binders, the characteristic peak positions were not significantly shifted, the peak shape was still sharp, and the baseline was stable, indicating that the interlayer distance of LDHs was not changed, and asphalt did not intercalate into the LDHs layers. The broadening and weakening of the diffraction peak shape may be mainly due to the LDHs being covered by asphalt binder [30].

**Figure 2:**
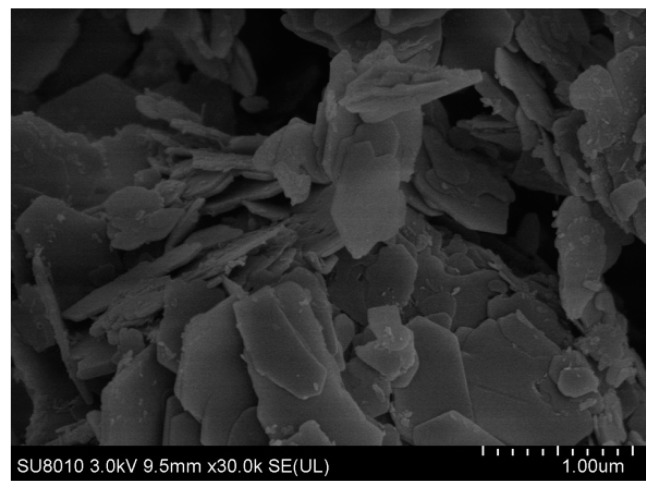
SEM image of layered double hydroxides (LDHs).

**Figure 3:**
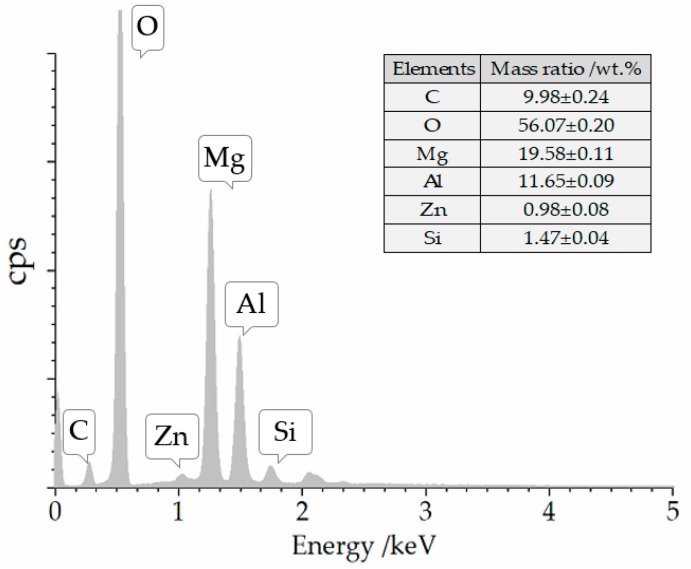
Energy-dispersive spectrometer (EDS) spectrum of LDHs.

SEM images of the ZnMgAl–CO_3_–LDHs are shown in Figure 2. A typical sheet structure with an average diameter of 1–2 μm and a thickness of approximately 50 nm can be seen. The main elements of the LDHs powder include C, O, Mg, Al, and a small amount of Zn and Si, as shown in Figure 3.

**Figure 4:**
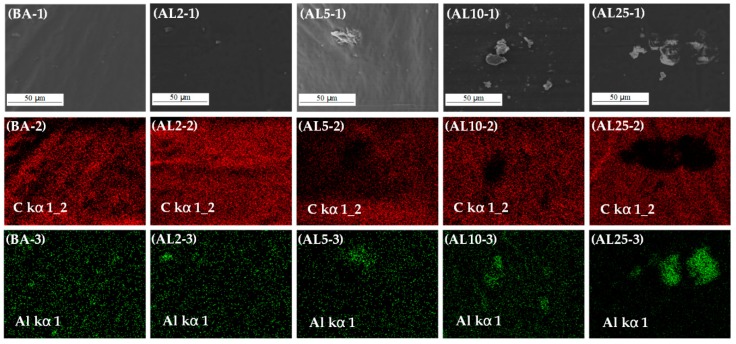
SEM images and EDS element mapping images (C in red and Al in green) of asphalt binders.

SEM can also capture the micromorphology of the asphalt binder surface [31]. To further investigate the dispersibility of LDHs plates inside asphalt binders, the binders were fully frozen in liquid nitrogen and fractured into pieces in a brittle manner. The fracture surface images are shown in the first row of Figure 4. A homogeneous structure can be seen on the surface of BA and AL2, whereas some fragments can be seen on the surface of AL5, AL10, and AL25. Next, EDS element mapping of these samples was used to identify the composition of the fragments. In Figure 4, the C signal (red images in second row) represents the asphalt binder, and the Al signal (green images in third row) represents the LDHs. The fragmented area shows more Al and less C, which means the LDHs aggregated there. Therefore, 2 wt.% ZnMgAl–CO_3_–LDHs can be evenly dispersed in asphalt binder, whereas an increase in ZnMgAl–CO_3_–LDHs increases the aggregation.

### 3.2. Flammability Characterization of Asphalt Binders

#### 3.2.1. LOI Tests

**Figure 5:**
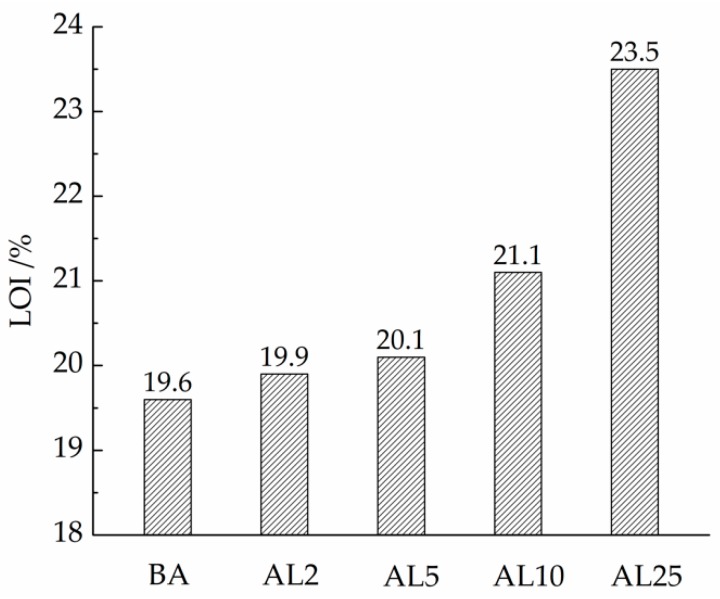
Limiting oxygen index (LOI) of asphalt binders.

LOI refers to the minimum oxygen concentration required to maintain a stable combustion of the sample. It is a key indicator of the flammability of a material under small flame conditions [32]. The higher the LOI of the material is, the harder it is to ignite it. As shown in Figure 5, the LOI of the base asphalt is only 19.6%, which is lower than 21%, so it can be easily ignited in air. With the addition of LDHs, the LOI of the asphalt binders increased slowly. The LOI of AL2 (asphalt binder with 2 wt.% LDH) was still only 19.9%. Due to the low radiation flux of the LOI test, the barrier mechanism of the LDHs cannot work well [32]; thus, 2 wt.% LDHs cannot effectively increase the LOI of the asphalt binder. With a dose of 25 wt.%, the LOI can reach 23.5%.

With the addition of LDHs, the dropping speed of the melted asphalt binder during the LOI test decreased. This is because the layered nanostructure of the LDHs hinders movement of the asphalt molecular chains, and the viscosity of the formed nanocomposites increases substantially.

#### 3.2.2. Cone Calorimeter Tests

The cone calorimeter test is one of the most ideal methods for characterizing the flammability characteristics of asphalt binder because of its good correlation with large-scale combustion test results [18]. The flammability characteristics of asphalt binders, including time to ignition (TTI), heat release rate (HRR), total heat release (THR), fire growth rate index (FIGRA), rate of smoke release (RSR), specific extinction area (SEA), and total smoke release (TSR) at a heat flux of 50 kW/m^2,^ are shown in Table 2.

**Figure 6:**
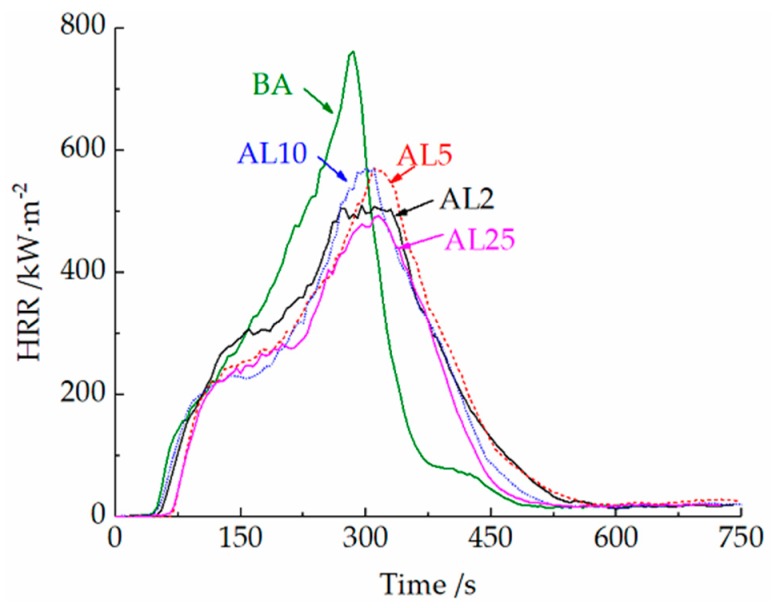
Heat release rate of asphalt binders.

As shown in Table 2, with an increasing LDH dose, the TTI of asphalt binders shows an increasing trend, whereas the THR gradually decreases. All HRR curves of asphalt binders with different LDH doses are shown in Figure 6. In Figure 6, the HRR curve of base asphalt (BA) shows a single peak, and the HRR reaches a peak of 761.7 kW·m^−2^ at 285 s. After the addition of LDHs, the HRR curves of asphalt binders show a shoulder at approximately 130–220 s, and the peak heat release rate (PHRR) shows a substantial reduction compared to that of the BA. Among them, the PHRR of AL2 with only 2 wt.% of LDHs is 33.0% lower than that of BA. This is probably due to the layered structure of the LDHs (as shown in Figure 1), which can block heat and mass exchange and promote the formation of barrier layers.

With the gradual increase of LDHs, the PHRRs of AL5 and AL10 show a slight increase. This is because the aggregation of LDHs under a relatively high dose limits the barrier effect described previously. If the dose further increased to 25 wt.%, the PHRR decreased again. The increased LDHs can work as a filler to dilute the combustible asphalt and absorb the heat of combustion by decomposition. The total heat release (THR) of AL25 also decreased by 21.0% compared to that of the BA.

The fire growth rate index (FIGRA) is defined as the ratio of peak time (*t*) to PHRR [33]. All of the binders with LDHs show a lower FIGRA compared to that of BA. Among them, the FIGRA of AL2 decreased by 35.3% compared to that of BA and that of AL25 further decreased by 9.5%.

**Figure 7:**
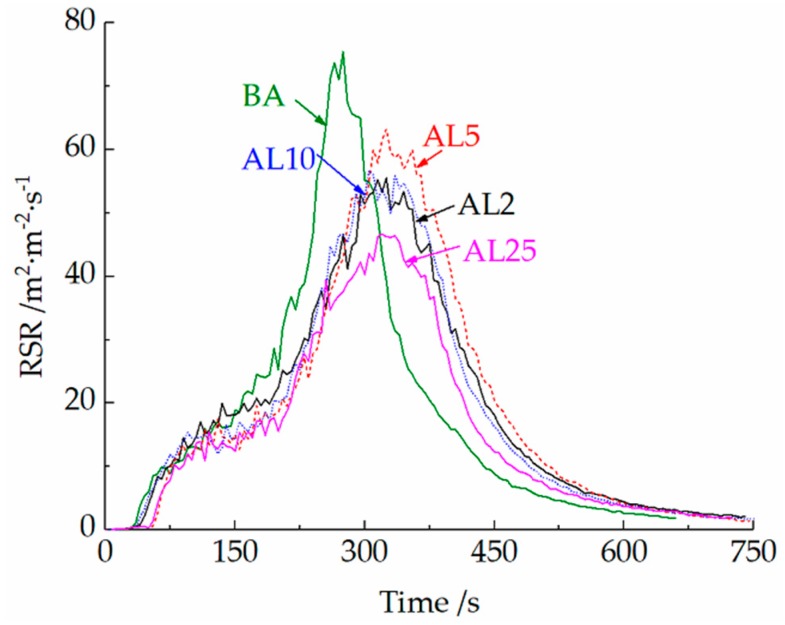
Smoke release rate of asphalt binders.

Approximately 85% of the deaths in a fire are caused by inhalation of toxic fumes, so the smoke release of asphalt combustion was also analyzed. With 2 wt.% and 25 wt.% LDHs, the PRSR of asphalt binder decreased by 26.9% and 38.2%, respectively, and the TSR decreased by 6.8% and 23.7%, respectively, compared to that of BA. The rate of smoke release (RSR) curves of different asphalt binders are shown in Figure 7. The RSR curves show a similar trend as the HRR curves because both the HRR and RSR are mainly controlled by the pyrolysis mass loss rate. It is worth noting that there are still some differences between smoke and heat release. The PHRR of AL5 and AL10 is nearly the same, whereas the PRSR of AL10 is 10.7% lower than that of AL5. The reduction of PRSR may result from the transition metal ions in the LDHs (Zn^2+^), which can catalyze the thermal degradation of the polymer [34], suppress the growth of polyaromatic hydrocarbons and the growth of their clusters, and reduce the formation of soot [35].

The specific extinction area (SEA) is the amount of smoke produced per unit mass loss. From Table 2, the SEA of asphalt binders decreases with the addition of LDHs, which indicates that the smoke release ability per unit binder combustion weakened. It can be concluded that the smoke suppression effect of the LDHs is due to reducing the combustion rate and the ability to release smoke.

### 3.3. Thermal Behaviors Analysis

**Figure 8:**
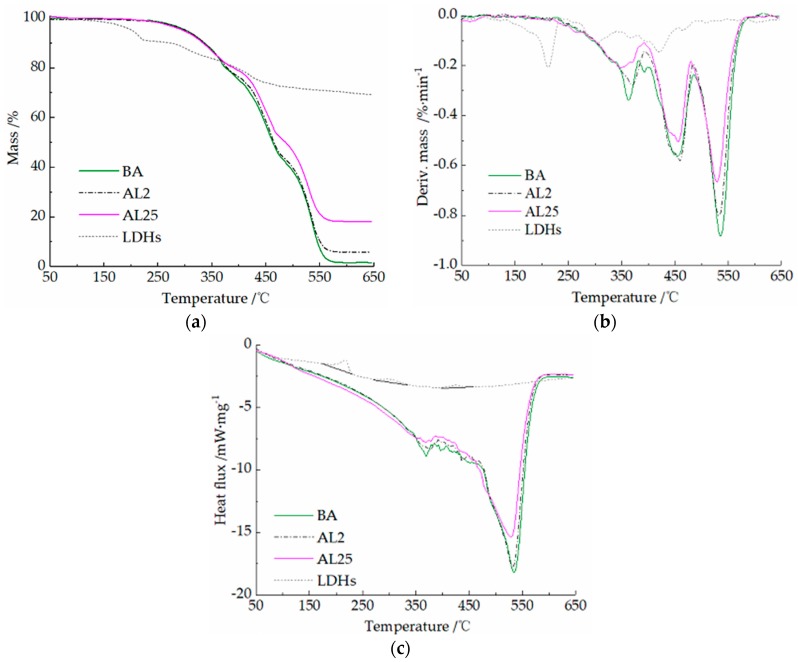
Thermogravimetry–differential scanning calorimetry (TG–DSC) curves of LDHs and asphalt binders: (**a**) TG, (**b**) derivative mass loss (DTG), and (**c**) DSC.

The TG–DSC curves reflect the mass change and heat absorption/release of the sample with a programmed temperature, which can help to determine the chemical reaction of the sample at different temperatures. The mass loss and heat flux curves of LDHs, BA, AL2, and AL25 are shown in Figure 8 to characterize the thermal stability of the samples and provide a basis for flame-retardant mechanism analysis.

From Figure 8a,b, the mass loss of three asphalt binders (BA, AL2, AL25) shows a similar trend; they all have three main combustion stages. The temperature ranges (TR), maximum mass loss rates (MMLR), and peak temperatures (PT) of each stage are shown in Table 3. The stages are divided by the valley of peaks in the derivative mass loss (DTG) curves.

With 2 wt.% of LDHs, the temperature range of stage I broadened, the maximum mass loss rate reduced from −0.339%·min^−1^ to −0.278%·min^−1^, and the peak temperature was also delayed. This is because the release of light components was delayed by the layered structures of the LDHs, and the longer residence time of light components favors the formation of polyaromatic structures during combustion. The residue ratio also increased from 3.6% to 7.9%.

Upon increased addition of the LDHs, the TR of AL25 shows no obvious change, and the thermal stability cannot be further increased. In addition, the MMLR of AL25 reduced, and the burning rate substantially decreased. This may be mainly because LDHs act as fillers and dilute a proportion of asphalt, and the decomposition of LDHs can absorb the heat of asphalt combustion.

From Figure 8a,b, the mass loss of LDHs had three main stages at 133–232 °C, 232–343 °C, and 343–493 °C, corresponding to the loss of loosely bound water in the interlayer space, –OH on the layer plates, and CO_3_^2−^ between these layers, respectively [36,37]. However, there are no mass loss peaks or endothermic peaks in the curves of AL25 combustion between 133 °C and 232 °C. This may be due to the loss of loosely bound water of LDHs during the blending process of the asphalt binders. The TRs for stages II and III of LDHs decomposition correspond to stages I and II of asphalt combustion, so the heat release of asphalt combustion is reduced, as shown in Figure 8c.

### 3.4. Combustion Residues Analysis

**Figure 9:**
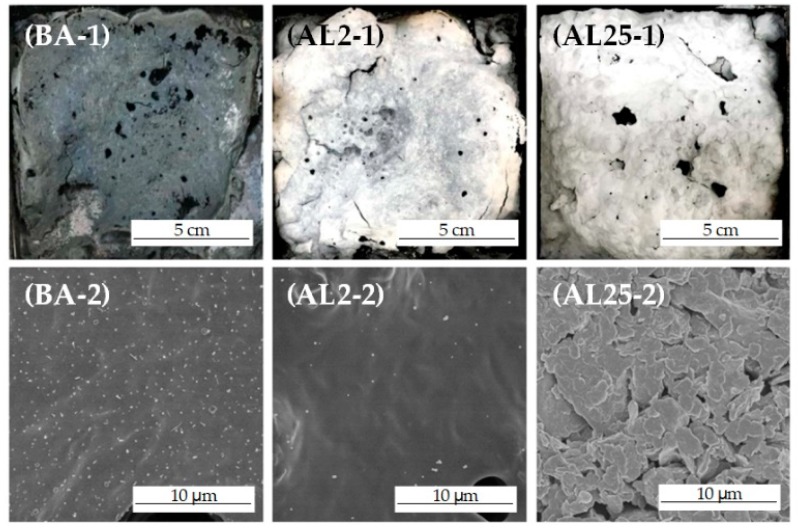
Digital and SEM images of the residues after cone calorimeter tests.

The formation of a barrier layer is one of the most important mechanisms that influences the flame retardancy of asphalt binders. The digital and SEM images of the residues after cone calorimeter tests are shown in Figure 9. It can be seen that the combustion residues of AL2 and AL25 are more complete than those of BA in the digital images in the first row of Figure 9. The asphalt binder with LDHs can form a more complete residue shell with fewer holes on the surface, indicating that LDHs can improve the integrity of the residue.

In can be seen in the SEM images in the second row of Figure 9 that there are many small pores in the residue on the surface of the BA, which was mainly caused by the escape of light gases that were produced by pyrolysis during the burning process. The residue surface of AL2 is flat and without cracks; it can inhibit the emission of combustible gases and prevent transmission of heat and oxygen. The well-dispersed LDHs can also prevent the collapse of the char layer, thus improving the integrity of the residue and further reducing the burning rate of the asphalt. The residue of AL25 shows a sintered sheet structure, which may be formed by stacking a large number of collapsed structures after the decomposition of the LDHs. Due to the poor dispersion of the LDHs, large amounts of them cannot contribute to the formation of a dense barrier layer.

**Figure 10:**
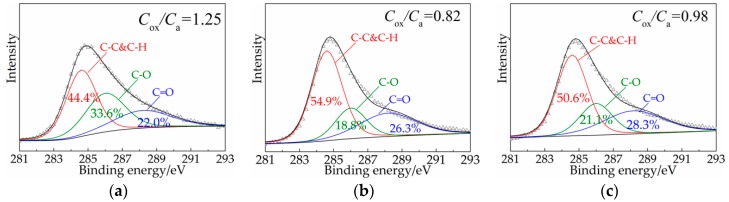
C 1s XPS spectra of the residues after cone calorimeter tests: (**a**) BA, (**b**) AL2, and (**c**) AL25.

Figure 10 shows the C 1s spectra of the residues after the cone calorimeter tests. The binding energies for the three bands are at approximately 284.6 eV, 286.0 eV, and 288.2 eV, which were contributed by C–H and C–C bonds in aliphatic and aromatic species, C–O bonds in ether linkages, and C=O groups in carbonyl, respectively [38]. The increase in C–C and C–H content indicates that a more polyaromatic structure was formed during combustion. Polyaromatic structures are favorable for increasing the compactness and thermal oxidation resistance of the char layer.

The content of oxidized carbons (C–O/C=O) and aliphatic/aromatic carbons (C–C/C–H) is represented by *C*_ox_ and *C*_a_, respectively. The thermal oxidative resistance of the char layer can be investigated by calculating *C*_ox_/*C*_a_. The smaller the *C*_ox_/*C*_a_ is, the greater the proportion of oxidized carbons in the residue. Figure 10 shows that LDHs can reduce the *C*_ox_/*C*_a_ value of the char layer. The addition of 2 wt.% LDHs reduces the *C*_ox_/*C*_a_ value by 0.43, and the addition of 25 wt.% LDHs reduces the *C*_ox_/*C*_a_ value by 0.27. These results indicate that LDHs are beneficial for improving the thermal oxidation resistance of asphalt residues. With 2 wt.% LDHs, the char layer shows the highest thermal oxidation resistance and compactness of the LDHs contents considered in this study.

## 4. Conclusions

The flame-retardant mechanism of LDHs on asphalt binders was investigated in this study, and the following conclusions can be drawn:

1. A small amount of LDHs (2 wt.%) can decrease the peak heat and smoke release rate of LDHs in cone calorimeter tests. The layered structures can suppress the release of light components and favor the formation of polyaromatic structures during combustion, thus improving the oxidation resistance and compactness of the char layer. Well-dispersed LDHs can also prevent the collapse of the char layer, thus improving the integrity of the residue.
2. A content of 25 wt.% LDHs can further increase the LOI of asphalt binder and decrease the heat and smoke release during combustion. The decomposition of LDHs can absorb the heat release of the initial two stages of asphalt combustion and reduce the burning rate of asphalt. Due to the loss of loosely bound water from the LDHs during the blending process and the decrease of dispersibility at a high LDH dose, the improvement of thermal stability was limited.

## Figures and Tables

**Table 1:** Basic properties of the base asphalt binder.

Properties		Standard	Test Results
Penetration at 25 °C/0.1 mm		ASTM D5-06	65.8
Ductility at 10 °C/cm		ASTM D113-07	42.0
Softening point/°C		ASTM D36-06	47.8
Flash point/°C		ASTM D92-05	340
Viscosity at 60 °C/Pa·s		ASTM D4402-06	193
Element content/wt.%	C	ASTM D5373	84.4
	H	ASTM D5373	10.9
	N	ASTM D5373	0.5
	S	ASTM D5373	2.7
	O	ASTM D5373	0.9
SARA fraction/wt.%	Saturates	ASTM D4124-09	21.1
	Aromatics	ASTM D4124-09	50.2
	Resin	ASTM D4124-09	20.7
	Asphaltenes	ASTM D4124-09	7.9

**Table 2:** Flammability characteristics obtained from cone calorimeter tests.

Samples	TTI	HRR (kW·m^−2^)		FIGRA	THR	PRSR	SEA	TSR
	(s)	Peak	*t* (s)	(kW·m^−2^·s^−1^)	(MJ·m^−2^)	(m^2^·m^−2^·s^−1^)	(m^2^·kg^−1^)	(m^2^·m^−2^)
BA	29	761.7	285	2.67	146.1	75.8	3004	14750
AL2	33	510.4	295	1.73	135.3	55.4	2825	13744
AL5	40	570.2	310	1.84	133.1	63.3	2823	14171
AL10	36	570.7	300	1.90	127.1	56.6	2702	13479
AL25	47	493.2	315	1.57	115.5	46.4	2636	11265

**Table 3:** Characteristic parameters of TG analysis of BA, L2, L25, and LDHs.

Samples	Stage I			Stage II			Stage III			Residue Ratio /%
	TR /°C	MMLR /%·min^−1^	PT /°C	TR /°C	MMLR /%·min^−1^	PT /°C	TR /°C	MMLR /%·min^−1^	PT /°C	
BA	264–380	−0.339	363	380–486	−0.564	454	486–600	−0.883	534	3.6
L2	260–393	−0.278	369	393–485	−0.581	459	485–600	−0.800	532	7.9
L25	256–391	−0.209	346	391–480	−0.505	456	480–587	−0.666	529	20.3
LDHs	133–232	−0.206	212	232–343	−0.110	305	343–493	−0.145	420	73.3
